# Breast Cancer Characteristics and Outcomes in Canadian Black Women by Ancestry

**DOI:** 10.3390/curroncol32110616

**Published:** 2025-11-04

**Authors:** Anna N. Wilkinson, Aisha Lofters, Moira Rushton, Jean M. Seely, Carmina Ng

**Affiliations:** 1 Department of Family Medicine, University of Ottawa, Ottawa, ON K1H 8L6, Canada; 2 Peter Gilgan Centre for Women’s Cancers, Women’s College Hospital, Toronto, ON M5S 1B2, Canada; aisha.lofters@wchospital.ca; 3 The Ottawa Hospital Cancer Centre, University of Ottawa, Ottawa, ON K1H 8L6, Canada; moirushton@toh.ca; 4 Department of Radiology, University of Ottawa, Ottawa, ON K1H 8L6, Canada; jeseely@toh.ca; 5 Centre for Population Health Data, Statistics Canada, Government of Canada, Ottawa, ON K1A 0T6, Canada

**Keywords:** breast cancer, race, ethnicity, cancer registry, outcomes, women’s health, black women

## Abstract

Breast cancer is the most common cancer among women in Canada. Its presentation and outcomes vary significantly by race/ethnicity. This study explores breast cancer incidence, age at diagnosis, stage, subtype, and mortality, comparing Black and White women aged 20 years and older, using the 2011 and 2016 Canadian Census Health and Environment Cohorts databases. Black women were disaggregated into Caribbean, Central/West African (C/WA), Southern/East African (S/EA), and “Other” ancestry groups. The Black female study population had a lower mean age (43.0 years) than the White (50.5 years). Black women had lower overall age-standardized breast cancer incidence than White women. The age-specific incidence in Black women ages 30–39 of Caribbean origin was higher (RR 95% CL, 1.36, 1.04–1.79; 58.7 vs. 43.1 cases/100,000 person-years) than in White. White women had 14.6% of cases diagnosed at ages 20–49 compared to over 50% in Black women of C/WA and S/EA origins, with highest proportions of diagnoses occurring at least 10 years earlier among Black women (C/WA 46, S/EA 48, Caribbean 57, White 67). Proportions of prognostic stage I diagnoses were less common among Black vs. White women (53.2% vs. 65.9%, *p* < 0.0001), and triple negative breast cancer was more frequent among Black women (17.1% vs. 9.9%, *p* < 0.0001), particularly those of Central/West African ancestry (21.8%). Higher age-specific mortality was observed among Black women with Caribbean origins aged 40–49 (RR 95% CL, 1.70, 1.19–2.42) and 50–59 (RR 95% CL, 1.42, 1.08–1.88) compared to White women. Breast cancer characteristics and outcomes vary substantially by ancestry within Canada’s Black population. Tailored screening strategies accounting for earlier onset and aggressive subtypes may help mitigate disparities.

## Simple Summary

Breast cancer characteristics vary by race/ethnicity and ancestry in Canada. Using population-based census cohorts, this study compared Black and White women diagnosed with breast cancer aged 20 and older. Many Black women were diagnosed with breast cancer at younger ages, with over half of cases in Central/West and Southern/East African women occurring before age 50 compared to only one in seven cases in White women. Black women were more often diagnosed at stage II or higher and more commonly had triple negative breast cancer. The presence of the triple negative subtype was very high among Black women with Central/West African origins, consistent with patterns linked to West African genetic ancestry. Age-specific mortality was higher among Black women with Caribbean origins in their 40s and 50s. These findings highlight the substantial variation of breast cancer burden across Black ancestry groups in Canada and suggest that a greater awareness is needed and that earlier, tailored screening strategies may reduce disparities.

## 1. Introduction

One out of every eight women in Canada will experience a breast cancer diagnosis over her lifetime [1]. Breast cancer research has historically reflected epidemiology and outcomes in White women. There is an increasing appreciation that breast cancer is not a uniform disease; rather, characteristics and outcomes differ across race and ethnicities. Breast cancer is especially unique in Black women, with differences noted in subtype and mortality compared to cases in White women [2].

However, race is a social construct, not a biological or genetic category, and it is not clear that all Black women are at equal risk [3]. In 2021, there were 1.5 million Black individuals in Canada, accounting for 4.3% of the population. The Black population in Canada is diverse, with multi-generational communities established through pre-Confederation transatlantic settlement and more recent migration from the Caribbean and Africa. Approximately 4 in 10 Black individuals were born in Canada, while over half the Black population are immigrants, with 182 different countries of birth. Immigration patterns have changed over time, with Black immigration from 1960 to 1990 coming mostly from the Caribbean and after 1990 shifting to be primarily from Africa. African-born Black populations in Canada increased by 531% from 1996 to 2021, rising from 14.0% to 32.6% of the total Black population. At the same time, the proportion of the Black population from the Caribbean dropped from 38.0% to 21.0%. The Black population has a median age of 30.2 compared to 41.2 for the total Canadian population, and nearly three-quarters of Black individuals are younger than 45 [4].

There has been little research investigating breast cancer in Black women in Canada. Previous work by our team has shown that Black women have the highest proportion of cases being diagnosed at age 50 compared to 65 in White women [2]. Although Black women have a lower breast cancer incidence than White women, they are significantly more likely to be diagnosed with advanced-stage disease and have higher rates of aggressive triple negative breast cancer than White women [5]. Mortality in Black women aged 40–49 years is 40% higher than in White women in the same age group, and the highest proportion of breast cancer deaths for Black women occurs at age 56 compared to 79 among White women. To date, there are no studies that disaggregate Canadian Black populations to examine differences in breast cancer outcomes according to ancestry.

This study uses linkages between census, cancer and death data to allow disaggregation of the Canadian Black populations by ancestry and examine breast cancer characteristics in these groups and in comparison to White women.

## 2. Methods

### 2.1. Data Sources

This is a retrospective population-based cohort study using the 2011 and 2016 Canadian Census Health and Environment Cohorts (CanCHECs) [6]. As nationally deidentified data collected by Statistics Canada were used, ethics approval was not required.

The 2011 and 2016 CanCHECs are linked databases combining information from the 2011 National Household Survey (NHS) and the 2016 long-form Census with the Canadian Cancer Registry (CCR) (maximum follow-up to 2021) and the Canadian Vital Statistics Deaths Database (CVSD) (maximum follow-up to 2023).

Due to lack of cancer data for certain jurisdictions at the time of linkage, maximum cancer incidence follow-up for residents of Québec, Nova Scotia, and Newfoundland and Labrador (2016 cohort only) ends 31 December 2017, 2019, and 2020, respectively.

Death data were linked to the 2016 cohort for deaths up to 31 December 2023, and up to 31 December 2022 for the 2011 cohort. Due to lack of death data for Yukon at the time of linkage, maximum mortality follow-up ends 31 December 2016 for these residents. Females were identified by the “sex” variables available in the cancer registry and/or death record and/or census or NHS, referring to sex at birth. Only the first breast cancer case during follow-up for each individual was considered. SAS version 9.4 was used for all analyses.

### 2.2. Study Population

The 2011 and 2016 cohorts were pooled, with duplicate cohort members removed. Race/ethnicity and ancestry information were derived using the population group and ethnic or cultural origin concepts common to both the 2011 NHS [7,8] and 2016 Census [9,10]. Females aged 20 and older on census/NHS day who self-identified as “Black” (*n* = 135,665) or “White” (*n* = 3,918,405) for the population group question were included for incidence and mortality follow-up. Based on the question, “What were the ethnic or cultural origins of this person’s ancestors?”, Black women were further categorized by their reported origins as Caribbean (*n* = 70,240), Central/West African (C/WA, *n* = 19,485), and Southern/East African (S/EA, *n* = 18,355), with any North African and other origins reported by Black women grouped into “Other” (*n* = 27,585) origins (Table S1). As up to six origins could be reported, the first reported Caribbean or African ancestry was used for those who reported more than one Caribbean and/or African origin. The 2011 and 2016 cohorts were pooled in order to increase the coverage and sample size for the Black female population, as analyzing the cohorts separately would not yield sufficient statistical power.

### 2.3. Incidence and Mortality Rates

Age-standardized incidence rates (ASIR) and age-standardized mortality rates (ASMR) per 100,000 person-years were calculated using the World Health Organization’s (WHO) world standard population. Person-years were accumulated from NHS/census day (May 10) to date of first breast cancer diagnosis (for incidence, max 10.6 years), breast cancer death (for mortality, max 11.6 years), date of death, or end of follow-up, whichever was earliest. Age-specific incidence and mortality rates were calculated for ages 30–39, 40–49, 50–59, 60–69, 70–79, and 80+. Rates, and rate ratios (RR), and 95% confidence limits (CL) were calculated with “White” as the reference category.

### 2.4. Age at Breast Cancer Diagnosis and Death

Mean, median, and peak age at breast cancer diagnosis and breast cancer death were calculated. The age distributions of diagnoses and deaths were plotted. To calculate peak ages at diagnosis and death, defined as the ages at which most diagnoses/deaths are estimated to occur among each group, the age distributions were smoothed using local regression (loess), and the peak ages were identified as the highest proportions. Differences in proportions at ages 20–39, 40–49, and 50+ were compared using two-sided chi-square tests.

### 2.5. Anatomic/Prognostic Stage and Molecular Subtypes

Information to determine stage and molecular subtypes was only consistently available on the CCR since diagnosis year 2012 for all jurisdictions except Québec (all years), Saskatchewan (for 2018 only), Nova Scotia (for 2019 only), Newfoundland and Labrador (for 2021 only), and Ontario (for 2021 only). Consequently, these cases and all cases diagnosed before 2012 were excluded from stage and subtype analyses. To facilitate comparisons with previous studies using anatomic stage, both anatomic and clinical prognostic stage at diagnosis were derived per the American Joint Committee on Cancer staging manual, 8th edition, using tumor, node, metastases, grade, hormone receptor (HR), and human epidermal growth factor 2 (HER2) status [11]. Anatomic stage describes the physical extent of the cancer, while prognostic stage incorporates biological factors, such as HER2 and HR status. For cases where clinical histologic grade was missing, pathological grade was used if available. Stages II, III, and IV are reported together, as low case numbers prevent reporting of these stages independently. Molecular subtypes were categorized as follows: hormone positive (HR+/HER2−), HR+/HER2+, HER2+ (HR−/HER2+) and triple negative (HR−/HER2−). Small sample sizes among the Black female groups and coverage gaps limited the ability to calculate age-specific and age-standardized rates for stage and subtypes. Differences in proportions of stage and subtypes were compared using two-sided chi-square tests. *p* < 0.05 was considered statistically significant.

## 3. Results

### 3.1. Incidence

There were 57,435 breast cancer cases, 1135 among Black women (Caribbean *n* = 690, Other *n* = 250, C/WA *n* = 100, S/EA *n* = 95) and 56,300 among White women during follow-up (Table 1). Mean follow-up time was 6.7 y (White 6.7 y, Black Caribbean 6.3 y, C/WA 5.8 y, S/EA 6.8 y, and Other origins 6.7 y). Black women consistently had lower ASIRs compared to White women (Table 2). However, Black Caribbean women 30–39 had a higher age-specific incidence rate than White women (RR 95% CL, 1.36, 1.04–1.79) with 58.7 cases per 100,000 person-years (95% CL 43.1–74.4) versus 43.1 cases per 100,000 person-years in White women (95% CL 41.1–45.1) (Table 3). Lower age-specific incidence rates were observed for Black Caribbean women 60–69 (RR 95% CL, 0.77, 0.66–0.90), 70–79 (RR 95% CL, 0.76, 0.64–0.91); Black women of other origins 40–49 (RR 95% CL, 0.72, 0.53–0.98), 60–69 (RR 95% CL, 0.75, 0.58–0.97); and Black women of S/EA origins 50–59 (RR 95% CL, 0.59, 0.41–0.84) compared to White women. Where sufficient data were available, age-specific incidence rates progressively increased with age among Black women, and among White women, the age 80+ rate was lower than that at ages 70–79.

### 3.2. Age at Diagnosis

**Figure 1:**
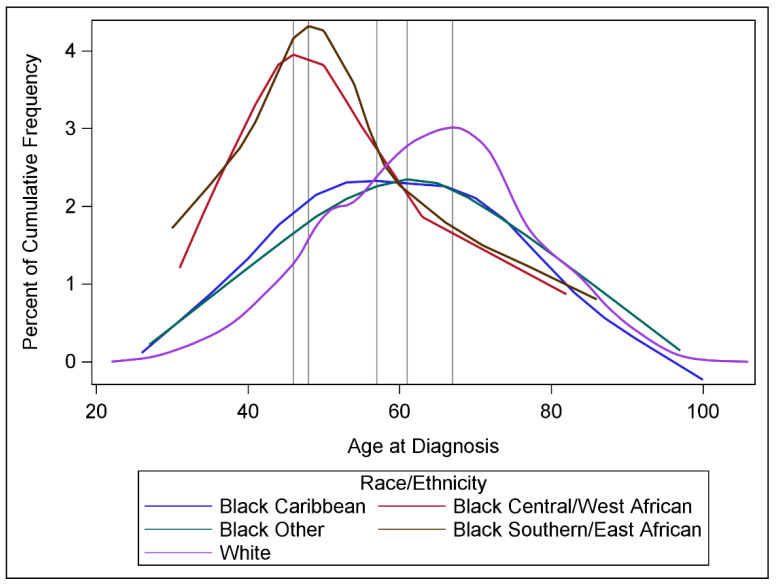
Distributions of age at breast cancer diagnosis, by race/ethnicity. Vertical lines denote the age at which highest percentage of cases were diagnosed, after smoothing.

The mean age of Black women of all origins was 43.0 years, ranging from 37.0 years among those with C/WA origins to 45.5 years among those with Caribbean origins, compared to 50.5 years for White women. Black women had an earlier peak age at diagnosis compared to White women (Table 1, Figure 1), with the peak ages at diagnosis consistently younger for Black women of C/WA (46), S/EA (48), Caribbean (57), and other origins (61), compared to 67 for White women. While 14.6% of breast cancer cases were diagnosed at ages 20–49 among White women, over 50% of cases were diagnosed at ages 20–49 for Black women of C/WA (54.2%) and S/EA (53.2%) origins. About one in four cases were diagnosed at ages 20–49 among Black women of Caribbean (26.6%) and other origins (23.2%). Only 3.5% of cases among White women were diagnosed at ages 20–39, compared with 15.6% among Black women of S/EA, 14.1% of C/WA, 9.0% Caribbean, and 6.7% of other origins.

### 3.3. Stage

Among 41,725 cases in scope for stage analyses, 1.6% (*n* = 680) were categorized as stage 0 and were excluded from further analyses. Anatomic stage could not be assigned for 5.7% (*n* = 2365, White 5.7%, Black Caribbean 6.1%, C/WA 4.9%, S/EA 5.1%, and other origins 8.2%) of cases, while prognostic stage could not be assigned for 13.0% (*n* = 5405, White 12.9%, Black Caribbean 17.4%, C/WA 15.9%, S/EA 11.4%, and other origins 20.7%) of cases. Cases with unknown stage were excluded.

**Figure 2:**
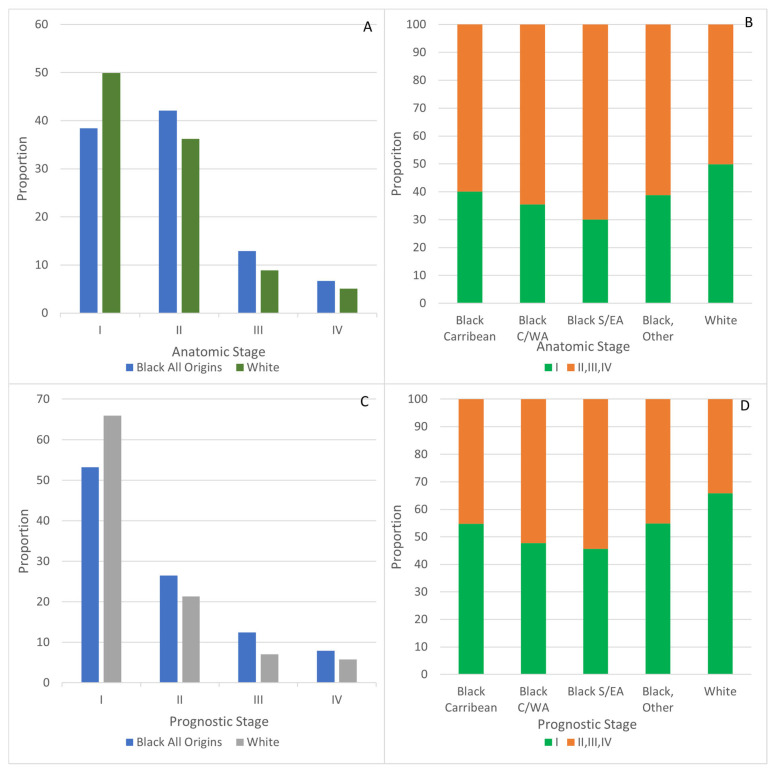
Stage at breast cancer diagnosis for Black and White women. (**A**) Anatomic, all stages, (**B**) Anatomic, Stage I compared to II, III, IV. (**C**) Prognostic, all stages. (**D**) Prognostic, Stage I compared to II, III, IV.

Approximately half of breast cancer cases among White women were diagnosed at anatomic stage I (49.9%) compared with 38.4% (*p* < 0.0001) of cases among Black women overall (Figure 2A). Considering anatomic stage I proportions by ancestry, they ranged from 30.1% in Black women of S/EA, 35.5% in C/WA, 38.8% in other and 40.1% in Caribbean women (Figure 2B).

Among cases diagnosed at ages 20–49, the proportion of breast cancer cases diagnosed at anatomic stages II, III, and IV was greater among Black women of all origins than that noted in White women (73.8% vs. 63.7%, *p* = 0.0014), and was 74.8% (*p* = 0.0132) among Black women of Caribbean origins.

Using prognostic staging, the proportion of stage I cases in White women was 65.9%, while it was 53.2% among Black women (*p* < 0.0001) (Figure 2C). Proportions of prognostic stage I ranged from 45.6% among Black women of S/EA origins and 47.8% for those with C/WA origins to 54.8% for Caribbean and 54.9% for those with other origins (Figure 2D).

Among cases diagnosed in younger women ages 20–49, a higher proportion were diagnosed at prognostic stages II, III, and IV among Black women overall compared with White women (60.1% vs. 45.3%, *p* < 0.0001). Among Black women, the proportions of prognostic stages II, III, and IV diagnoses ranged from 67.6% for those of S/EA and 62.4% for those of Caribbean origins to 48.6% for those with C/WA and 57.1% for those with other origins.

### 3.4. Molecular Subtype

**Figure 3:**
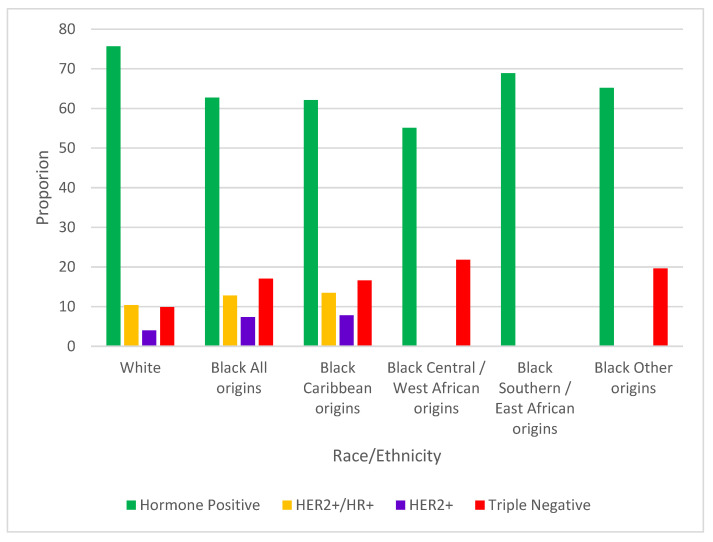
Proportions of breast cancer molecular subtypes, by race/ethnicity. Subtypes with fewer than 15 cases suppressed.

Among 41,725 cases in scope for subtype analyses, 6.5% (*n* = 2720, White 6.5%, Black Caribbean 5.6%, C/WA 4.9%, S/EA 6.3%, other origins 14.1%) could not be assigned a subtype and were excluded. The triple negative subtype was more common among Black women overall (17.1%) compared with White women (9.9%, *p* < 0.0001) (Figure 3). Among cases diagnosed at ages 20–49, the proportions of triple negative cases were not statistically different between Black and White women (Black 15.6% vs. White 13.1%, *p* = 0.2447). However, Black women had 29.0% of triple negative cases diagnosed at ages 20–49 compared to 19.4% among White women (*p* = 0.0097). Looking at cases diagnosed at ages 50 and over, the proportion of triple negative breast cancer was almost double among Black (18.0%) vs. White (9.4%, *p* < 0.0001). Approximately one-fifth of cases among Black women of C/WA (21.8%) and of other origins (19.6%) were triple negative, while 16.6% of cases were classified as such among Black Caribbean women. There were insufficient cases of triple negative breast cancer cases among Black S/EA women for analysis.

Among White women, 4.0% of cases were HER2+, whereas HER2+ breast cancer constituted 7.4% of cases among Black women overall (*p* < 0.0001). Among cases diagnosed at ages 20–49, Black women had a higher proportion of HER2+ cases than White women (Black 9.5% vs. White 6.0%, *p* = 0.0392). Among Black women, 41.1% of HER2+ cases were diagnosed at ages 20–49 compared to 22.1% among White women (*p* = 0.0018). A significantly higher proportion of HER2+ cancer was also seen in Black women diagnosed at age 50 and older compared to White women (Black 6.4% vs. White 3.7%, *p* = 0.003). There was no significant difference in the HR+/HER2+ subtype breast cancer proportions between Black and White women regardless of age at diagnosis. Comparisons for these two subtypes could not be made by ancestry, as not every ancestry category had adequate cases.

Three-quarters of cases were hormone receptor positive in White women (75.7%), while 62.7% of cases were this subtype in Black women (*p* < 0.0001). The proportions of hormone receptor positive cases were significantly higher in White women than Black women in cancers diagnosed at ages 20–49 (Black 56.8% vs White 64.7%, *p* = 0.0137) and at age 50 and above (Black 65.4% vs. White 77.6%, *p* < 0.0001). Proportions of hormone positive cases were 55.1%, 62.1%, 65.2%, and 68.9% among Black women of C/WA, Caribbean, other, and S/EA origins, respectively.

### 3.5. Mortality

There were 15,580 breast cancer deaths, 350 among Black (Caribbean *n* = 210, Other *n* = 80, C/WA *n* = 25, S/EA *n* = 35) women and 15,230 among White women during follow-up (Table 1). Mean follow-up time was 9.3 y (White 9.3 y, Black Caribbean 9.1 y, C/WA 9.0 y, S/EA 9.0 y, and other origins 9.1 y). ASMRs were not statistically different among Black groups compared with White (Table 4). Sufficient mortality data were only available for some age groups among Black women of Caribbean and those with other origins. Higher age-specific mortality rates were observed among Black women of Caribbean origins at ages 40–49 (RR 95% CL, 1.70, 1.19–2.42, 23.4 deaths per 100,000 person-years) and 50–59 (RR 95% CL, 1.42, 1.08–1.88, 43.1 deaths per 100,000 person-years) compared to White women (13.8 deaths among ages 50–59 and 30.3 deaths among ages 60–69 per 100,000 person-years) (Table 5). The peak age at death from breast cancer was lower for Black women of S/EA (53), C/WA (54), Caribbean (55), and other origins (66) compared to 71 for White women. For Black and White women, age-specific mortality rates were highest among ages 70–79 (Black Caribbean 65.0, Black other origins 92.2, White 81.8 deaths per 100,000 person-years) and 80+ (Black Caribbean 150.1, Black other origins 221.6, White 171.6 deaths per 100,000 person-years).

## 4. Interpretation

This is the first Canadian study to disaggregate Black populations and explore breast cancer outcomes based on ancestry. Black women had a markedly lower peak age at diagnosis than White women, and Black women of C/WA and S/EA origins had over half of breast cancer cases diagnosed before age 50 and about one in seven cases diagnosed below age 40. This is in part due to the younger population age structure among the Black women. Although the overall incidence of breast cancer was lower in Black women in comparison to White, Black women of Caribbean origins aged 30–39 had a higher incidence of breast cancer than White women at this age. A lower proportion of cases were diagnosed at stage I among Black compared to White women across all ancestry groups, particularly among Black women of S/EA origins. Biologically aggressive triple negative breast cancer was more commonly diagnosed among Black women than White, particularly among Black women with C/WA origins, while the proportions of triple negative cases among those diagnosed at ages 20–49 were not significantly different between Black and White women. The peak age of death ranged from 5 to 18 years younger in Black than White women, and although overall breast cancer mortality in Black women was not elevated compared to White, Black women of Caribbean origins had 70% and 42% higher mortality rates than White women at ages 40–49 and 50–59, respectively.

Analyzing Canadian Black populations by ancestry origins provides deeper insight into the underlying causes of the disparities in triple negative breast cancer. Triple negative breast cancer accounts for approximately one-third of breast cancer cases across the African continent, compared to about 10% among White women in Canada [12]. Within Africa, the highest rates of triple negative breast cancer are observed in West Africa, where it represents 45.7% of cases overall and up to 57.2% of cases in Ghana—and in one study, up to 83% of Ghanian cases [12]. In contrast, triple negative rates in East Africa are lower, comprising about a quarter of cases. The elevated prevalence of triple negative breast cancer in West African populations is particularly relevant given the historical and genetic ties between West Africa and the Black populations of North America. Between the 16th and 18th centuries, the transatlantic slave trade forcibly displaced large numbers of individuals from sub-Saharan and West Africa to the Americas and the Caribbean. It is estimated that up to 66% of the present-day Black population in the Americas traces its ancestry to West Africa [13]. This shared genetic lineage raises the possibility of founder mutations that contribute to triple negative breast cancer risk.

Among Canadian Black women of C/WA ancestry, 21.5% of cases were triple negative breast cancer, while a lower proportion (16.5%) was observed among Black women of Caribbean origins. There were insufficient cases among those with S/EA origins, indicating a low proportion of cases were triple negative. In comparison to Black women in the United States (US), a low proportion of triple negative cases were observed among those East-African born (12%), with similar proportions among those US-born (24%), West Africa-born (24%), and Caribbean-born (21%) [14]. These findings suggest that ancestral origin within the African diaspora may influence triple negative breast cancer risk, supporting the hypothesis that elevated triple negative rates in many Canadian-born Black women may be partly attributable to West African genetic heritage. Although triple negative rates are known to be higher in women with BRCA mutations, specifically *BRCA1*, it is not clear that this is the driver for increased triple negative rates in Black women of C/WA origins. A pathogenic BRCA variant has been noted in Senegalese women, which may reflect a founder mutation, as well as increased rates of BRCA mutations in individuals with breast cancer in the Bahamas and Ghana and *BRCA1* in Burkina Faso [15,16]. At the same time, other studies suggest lower rates of BRCA mutations in African American women [17]. Genetic profiling of African American women with breast cancer found that 22% had a clinically relevant mutation, suggesting that yet unknown genetic factors may be at play [18]. Compared to White women, African American, Caribbean, and Nigerian women have increased expression of the transcription factor Kaiso, which may facilitate tumor growth [19]. The microenvironment of breast cancers in Black women has been found to have a distinctive T-cell exhaustion despite a stronger overall immune response, suggesting that immunotherapy may be particularly effective in these women [20]. However, it appears that Black women, at least in the US, may be less likely than their White counterparts to receive immunotherapy and maybe more likely to decline treatments in part due to a longstanding distrust of the scientific community, related to past and present injustices [21,22].

Up to 18% of triple negative breast cancers may be misdiagnosed on mammograms, as they tend to present as a circumscribed mass that can simulate a benign lesion and may lack suspicious features such as calcifications, irregular shape, and spiculated margins [23,24]. Triple negative breast cancers are known to bypass the in situ phase and have a high growth rate, doubling every 7.5 months compared to the every 9 months seen in hormone receptor positive breast cancers. Thus, biennial mammography may not be adequate for timely diagnosis of this breast cancer subtype. Screening is essential for triple negative breast cancers, as those detected by screening have a significantly higher 5-year overall survival than those detected due to symptoms [25]. Modelling the relative contributions of mammographic screening to mortality reduction indicates that breast cancer screening accounts for 40% of the mortality reduction in triple negative breast cancers, compared to 19% in hormone positive breast cancers [26]. An additional complicating factor is that women from sub-Saharan Africa and North Africa remain at a higher risk of being underscreened, with one study showing screening uptake of only 35.3% in sub-Saharan African women from Muslim-majority countries [27]. Breast cancer screening recommendations for populations with high rates of triple negative breast cancer should consider risk factors including growth rates and mammographic sensitivity as well as screening participation to optimize outcomes.

Black women with Caribbean origins had more stage II/III/IV at diagnosis and, at ages 40–59, increased mortality. Caribbean women have been previously noted to be less likely to have a screen-detected cancer [28]. These findings may be because below the age of screening, which has been recommended in Canada to begin at age 50, breast cancers more likely would be symptomatically detected, translating into higher stage disease, and ultimately, increased mortality. Additionally, 29% of triple negative cases among Black women were diagnosed at ages 20–49, which could translate into increased mortality given elevated mortality within 5 years of diagnosis for this subtype [29]. Social determinants of health also could contribute to increased mortality, especially since Black women in the US have an increased breast cancer mortality rate of 38% across all age groups, while in Canada, with our single-payer health care system, we noted no significant difference in age-standardized mortality between Black and White women [30].

The peak age of diagnosis at 46 and 48 for Black women of C/WA and S/EA origins as well as the 36% higher rate of breast cancers in those with Caribbean origins in their 30s is important to note. These findings are congruent with other studies [5,13,30,31]. A younger population structure contributes to lower peak ages at diagnosis, as there are lower proportions of older people. With 30.4% of cases occurring before age 50, and 9.5% of cases diagnosed before age 40 in Black women, increased clinical awareness is essential. There must be greater awareness among women to seek evaluation for any breast changes regardless of age. Health care providers must appreciate the importance of earlier screening onset in these women and remain vigilant to the signs of symptomatic breast cancer in younger women.

This study was limited by sample size given that only those breast cancer cases diagnosed in individuals who had also completed the 2011 NHS and/or 2016 Census could be included. The CanCHEC databases were created based on probabilistic linkage strategies. Race, ethnicity, and ancestry information were self-reported at the time of completion of the NHS or census. These factors could lead to potential classification errors. Additionally, as certain data were unavailable from multiple provinces, these results may not represent all Canadians. Collection of race and ethnicity data within the Canadian Cancer Registry and improved completeness of stage and subtype information would facilitate these analyses in the future.

This study illustrates the heterogeneity of breast cancer characteristics and outcomes in Canadian Black populations. High proportions of triple negative breast cancer among Black women of C/WA ancestry likely reflect the lasting impact of the historical involuntary migration. The higher proportions of triple negative breast cancer, diagnostic stage disadvantage, and earlier age of onset in Canadian Black compared to White women, coupled with significantly increased mortality at younger ages, mandates an examination of barriers to care across the cancer continuum—from screening to diagnosis and treatment. Tailoring screening strategies, including appropriate age of screening onset, frequency, and modality, could represent a means to mitigate racial disparities in breast cancer outcomes [32].

## Figures and Tables

**Table 1:** Sample size, median, mean (SD) age of sample at start of follow-up, peak age at diagnosis and death for breast cancer cases, breast cancer deaths.

Race/Ethnicity	*N*	Median Age of Sample (y)	Mean Age of Sample (y)	Breast Cancer Cases	Median Age at Diagnosis (y)	Mean Age Diagnosis (y)	Peak Age at Diagnosis (y)	Breast Cancer Deaths	Median Age at Breast Cancer Death (y)	Mean Age at Breast Cancer Death (y)	Peak Age at Breast Cancer Death (y)
Black Caribbean origins	70,240	44	45.5	690	59	59.3	57	210	64	65.1	55
Black Other origins	27,585	42	44.1	250	61	61.0	61	80	71	66.4	66
Black Central/West African origins	19,485	35	37.0	100	48	49.2	46	25	48.5	50.6	54
Black Southern/East African origins	18,355	37	38.6	95	49	49.8	48	35	51	53.3	53
White	3,918,405	51	50.5	56,300	64	63.8	67	15,230	72	71.0	71

Source: Canadian Census Health and Environment Cohorts (CanCHEC) 2011 and 2016, Canadian Cancer Registry (CCR) follow-up to 2021, mortality follow-up to 2022 and 2023, respectively. SD: Standard Deviation. Counts randomly rounded to base 5 in accordance with Statistics Canada’s confidentiality guidelines.

**Table 2:** Age-standardized (world) breast cancer incidence rates and rate ratios (reference = White), by race/ethnicity.

Race/Ethnicity	Age-Standardized Incidence Rate (Per 100,000 Person-Years)	Rate Lower CL	Rate Upper CL	Age-Standardized Incidence Rate Ratio	Rate Ratio Lower CL	Rate Ratio Upper CL
Black Caribbean origins	123.1	113.7	133.4	**0.89**	**0.83**	**0.97**
Black Other origins	110.5	97.0	126.0	**0.80**	**0.71**	**0.91**
Black Central/West African origins	90.2	70.4	118.0	**0.65**	**0.52**	**0.83**
Black Southern/East African origins	74.4	58.2	95.3	**0.54**	**0.43**	**0.68**
White	137.9	136.6	139.1			

Source: Canadian Census Health and Environment Cohorts (CanCHEC) 2011 and 2016, Canadian Cancer Registry (CCR) follow-up to 2021. CL: Confidence Limit.

**Table 3:** Age-specific breast cancer incidence rates and rate ratios (reference = White), by race/ethnicity.

Race/Ethnicity	Cases	Age at Diagnosis Group	Crude Rate (Per 100,000 Person-Years)	Rate Lower CL	Rate Upper CL	Crude Rate Ratio	Rate Ratio Lower CL	Rate Ratio Upper CL
Black Caribbean origins	55	30–39	58.7	43.1	74.4	**1.36**	**1.04**	**1.79**
	125	40–49	130.5	107.3	153.8	0.93	0.78	1.11
	165	50–59	202.7	172.1	233.4	0.90	0.77	1.05
	150	60–69	258.6	217.5	299.7	**0.77**	**0.66**	**0.90**
	125	70–79	309.7	255.6	363.8	**0.76**	**0.64**	**0.91**
	60	80+	323.9	241.3	406.6	0.90	0.70	1.16
Black Central/West African origins	40	40–49	128.9	89.0	168.9	0.92	0.67	1.25
	30	50–59	176.0	114.1	238.0	0.78	0.55	1.11
Black Other origins	15	30–39	38.5	19.6	57.4	0.89	0.55	1.46
	40	40–49	101.4	70.4	132.5	**0.72**	**0.53**	**0.98**
	65	50–59	190.5	143.5	237.5	0.85	0.66	1.08
	55	60–69	251.9	186.5	317.3	**0.75**	**0.58**	**0.97**
	45	70–79	340.3	245.0	435.6	0.84	0.63	1.11
	30	80+	388.3	241.9	534.8	1.08	0.74	1.58
Black Southern/East African origins	15	30–39	41.7	20.6	62.8	0.97	0.58	1.61
	35	40–49	103.2	69.5	137.0	0.73	0.53	1.02
	30	50–59	132.7	86.0	179.5	**0.59**	**0.41**	**0.84**
White	1795	30–39	43.1	41.1	45.1			
	6255	40–49	140.6	137.1	144.1			
	12405	50–59	225.3	221.3	229.3			
	16515	60–69	335.8	330.7	340.9			
	12385	70–79	405.1	397.9	412.2			
	6790	80+	359.7	351.1	368.2			

Source: Canadian Census Health and Environment Cohorts (CanCHEC) 2011 and 2016, Canadian Cancer Registry (CCR) follow-up to 2021. CL: Confidence Limit. Groups with fewer than 15 cases suppressed.

**Table 4:** Age-standardized (world) breast cancer mortality rates, by race/ethnicity.

Race/Ethnicity	Age- Standardized Mortality Rate (Per 100,000 Person-Years)	Rate Lower CL	Rate Upper CL	Age-Standardized Mortality Rate Ratio	Rate Ratio Lower CL	Rate Ratio Upper CL
Black Caribbean origins	22.6	19.6	26.4	1.08	0.94	1.25
Black Other origins	13.6	7.6	25.1	0.65	0.39	1.09
Black Central/West African origins	22.5	17.7	29.0	1.08	0.86	1.35
Black Southern/East African origins	23.5	15.4	35.2	1.12	0.77	1.64
White	20.9	20.5	21.3			

Source: Canadian Census Health and Environment Cohorts (CanCHEC) 2011 and 2016, mortality follow-up to 2022 and 2023, respectively. CL: Confidence Limit.

**Table 5:** Age-specific breast cancer mortality rates, by race/ethnicity.

Race/Ethnicity	Age Diagnosis Group	Deaths	Crude Rate (per 100,000 Person-Years)	Rate Lower CL	Rate Upper CL	Crude Rate Ratio	Rate Ratio Lower CL	Rate Ratio Upper CL
Black Caribbean origins	40–49	30	23.4	15.3	31.5	**1.70**	**1.19**	**2.42**
	50–59	50	43.1	31.4	54.9	**1.42**	**1.08**	**1.88**
	60–69	35	41.6	28.0	55.1	0.86	0.62	1.19
	70–79	40	65.0	44.8	85.1	0.79	0.58	1.09
	80+	45	150.1	105.7	194.4	0.87	0.65	1.18
Black Other origins	50–59	15	41.5	22.9	60.2	1.37	0.87	2.15
	70–79	20	92.2	50.8	133.7	1.13	0.72	1.77
	80+	20	221.6	131.0	312.2	1.29	0.86	1.95
White	30–39	250	4.4	3.9	4.9			
	40–49	835	13.8	12.8	14.7			
	50–59	2250	30.3	29.0	31.5			
	60–69	3410	48.5	46.9	50.1			
	70–79	3730	81.8	79.2	84.4			
	80+	4735	171.6	166.7	176.5			

Source: Canadian Census Health and Environment Cohorts (CanCHEC) 2011 and 2016, mortality follow-up to 2022 and 2023, respectively. CL: Confidence Limit. Groups with fewer than 15 deaths suppressed.

## Data Availability

Data are available through the Research Data Centres program at Statistics Canada https://www.statcan.gc.ca/en/microdata/data-centres (accessed on 1 March 2025).

## Supplementary Materials

The following supporting information can be downloaded at: https://www.mdpi.com/article/10.3390/curroncol32110616/s1, Table S1: Ethnic origins included for Caribbean, Central or West African, and Southern or East African categories.
