# Supplementary material for: Breast Cancer Characteristics and Outcomes in Canadian Black Women by Ancestry
Source: Curr Oncol. 2025 Nov 4;32(11):616. doi: 10.3390/curroncol32110616 (PMC12651697; doi:10.3390/curroncol32110616)
Supplement: Supplementary file 1 [file curroncol-32-00616-s001.zip › curroncol-3849881-supplementary.docx]

| Supplementary Table1: Ethnic origins included for Caribbean, Central or West African, and Southern or East African categories. | | | | | |
| --- | --- | --- | --- | --- | --- |
|  |  |  |  |  |  |
| Caribbean | | Central or West African | | Southern or East African | |
| African Caribbean | | Akan, n.o.s. | | Afar |  |
| Antiguan | | Angolan | | Afrikaner | |
| Aruban |  | Ashanti | | Amhara | |
| Bahamian | | Bambara | | Bantu, n.o.s. | |
| Barbadian | | Bamileke | | Burundian | |
| Bermudian | | Baoulé |  | Djiboutian | |
| Carib |  | Beninese | | Eritrean | |
| Cuban |  | Burkinabe | | Ethiopian | |
| Dominica Islander | | Cameroonian | | Harari |  |
| Dominican | | Cape Verdean | | Hutu |  |
| Grenadian | | Central African | | Kenyan | |
| Guadeloupean | | Chadian | | Kikuyu |  |
| Haitian |  | Congolese | | Luo |  |
| Indo-Caribbean | | Edo |  | Malagasy | |
| Jamaican | | Esan |  | Malawian | |
| Kittitian/Nevisian | | Ewe |  | Mauritian | |
| Maroon | | Fante |  | Mozambican | |
| Martinican | | Fulani |  | Namibian | |
| Montserratian | | Ga-Adangbe | | Ndebele | |
| Puerto Rican | | Gabonese | | Nubian | |
| St. Lucian | | Gambian | | Oromo |  |
| Trinidadian/Tobagonian | | Ghanaian | | Réunionnais | |
| Vincentian | | Guinean | | Rwandan | |
| West Indian, n.o.s. | | Hausa |  | Seychellois | |
| Caribbean origins, nie. | | Igbo |  | Shona |  |
|  |  | Ivorian |  | Somali |  |
|  |  | Liberian | | South African | |
|  |  | Luba |  | South Sudanese | |
|  |  | Malian |  | Swahili |  |
|  |  | Malinké | | Tanzanian | |
|  |  | Mauritanian | | Tigrinya | |
|  |  | Mossi |  | Tswana (Batswana) | |
|  |  | Nigerian | | Tutsi |  |
|  |  | Nigerien | | Ugandan | |
|  |  | Senegalese | | Zambian | |
|  |  | Serer |  | Zimbabwean | |
|  |  | Sierra Leonean | | Zulu |  |
|  |  | Soninke | | Southern and East African origins, n.i.e. | |
|  |  | Togolese | |  |  |
|  |  | Wolof |  |  |  |
|  |  | Yoruba |  |  |  |
|  |  | Central and West African origins, n.i.e. | | | |
